# 

**Published:** 1908-01-25

**Authors:** 


### THE GRADUATES' MEDICAL SCHOOLS.

### Diary for the week January 27 to 31.

**THE POST-GRADUATE COLLEGE, West London Hospital, Hammersmith, W.**

**Lectures: At noon.**

**Jan. 27,** Dr. Low, **Pathological Demonstration.**

**Jan. 28 and 30,** Dr. Pritchard, **Practical Medicine.**

**At 5 p.m.**

**Jan. 27,** Mr. Bidwell, **Clinical, I.**

**Jan. 28,** Dr. Robert Jones, **Relation of Insanity to Allied Neuroses.**

**Jan. 29,** Dr. Beddard, **Practical Medicine, II.**

**Jan. 30,** Mr. Edwards, **Clinical, I.**

**Jan. 31,** Mr. R. W. Lloyd, **Anæsthetics, I,**

**MOUNT VERNON HOSPITAL POST-GRADUATE COURSE, Fitzroy Square.**

**At 5 p.m.**

**Jan. 30,** Dr. A. J. Whiting, **Aneurysm of the Thoracic Aorta.**

**THE THROAT HOSPITAL, Golden Square, W.**

**Lectures at 5.30 p.m.**

**Jan. 27,** Dr. H. W. F. Powell, **Acute Inflammatory Affections—Suppurative.**

**Jan. 30,** Mr. F. Rose, **Chronic Purulent Otitis Media.**

**CHARING CROSS HOSPITAL, Endell Street, W.C.**

**Post-Graduate Lecture at 4 p.m.**

**Jan. 30,** Mr. W. Hunter, **Medical.**

**LONDON SCHOOL OF CLINICAL MEDICINE, Seamen's Hospital, Greenwich.**

**At 4.0 p.m.**

**Jan. 27,** Dr. St. Clair Thomson.

**At 3.15 p.m.**

**Jan. 28,** Mr. Carless, **Movable Kidney.**

**At 2.15 p.m.**

**Jan. 31,** Dr. Rose Bradford, **Diabetes.**

**NATIONAL HOSPITAL FOR THE PARALYSED AND EPILEPTIC, Queen Square, Bloomsbury, W.C.**

**Lectures at 3.30 p.m.**

**Jan. 28,** Dr. A. Turner, **Cranial Nerve Paralysis.**

**Jan. 31,** Dr. J. Taylor, **Myopathies.**

**MEDICAL GRADUATES' COLLEGE AND POLYCLINIC, Chenies Street, W.C.**

**Lectures at 5.15 p.m.**

**Jan. 27,** Mr. James Sherren, **The Differential Diagnosis between Injuries of the Spinal Cord and those of Peripheral Nerves.**

**Jan. 28,** Dr. David Sommerville, **Mucous Colitis.**

**Jan. 29,** Dr. W. Langdon Brown, **Some Intestinal Intoxications.**

**Jan. 30,** Dr. Leonard Williams, **The Bogey of Albuminuria.**

According to the *Reichsmedizinal Kalender*, there were 31,416 medical men in Germany in 1907, a number which —as has been the case for several years—represents a moderate increase only over the preceding twelve months (1904, 30,091; 1905, 30,675; 1906, 30,931). This augmentation is expected to become more rapid during the next few years at least because medical students, who had previously been slightly declining in numbers, now show very appreciable increases from term to term. In Greater Berlin (with twenty-one suburbs) there are 3,418 doctors, or 11.7 per 10,000 inhabitants; in other large towns the ratio is 9.5, sinking to 2.9 per 10,000 inhabitants in the more thinly-populated country districts. The tendency to specialise is becoming more and more pronounced, specialists forming 20.2 per cent. of the whole medical fraternity. Of the several branches in which they practise, gynæcology accounts for the greatest number; then follow, in order of number, eye, ear, nose, and throat specialists, then surgeons.

THE HOSPITAL *January* 25, 1908.

*Name* ....................

*Address* ....................

....................

This Coupon must accompany manuscript or contributions intended for The Hospital.

